# Auditory results from cochlear implants in elderly people

**DOI:** 10.1590/S1808-86942010000400008

**Published:** 2015-10-19

**Authors:** Valéria Oyanguren, Maria Valéria Goffi Gomes, Robinson Koji Tsuji, Ricardo Ferreira Bento, Rubens Brito Neto

**Affiliations:** a Specialist in audiology, speech therapist of the cochlear implant group at the Clinical Hospital, Medical School, Sao Paulo University (HCFMUSP); b Doctoral degree, speech therapist of the otorhinolaryngology clinic division, HCFMUSP, coordinator of the speech therapy team of the cochlear implant group, HCFMUSP; c Doctoral degree, otorhinolaryngologist, HCFMUSP, coordinator of the cochlear implant group, HCFMUSP; d Full professor of the otorhinolaryngology discipline, Medical School, Sao Paulo University (FMUSP), head of the ophthalmology and otorhinolaryngology department, FMUSP; e Associate professor, collaborator in the otorhinolaryngology discipline, FMUSP, assistant physician in the otorhinolaryngology division, Clinical Hospital, Sao Paulo University

**Keywords:** health of the elderly, hearing, cochlear implants

## Abstract

According to data from the Brazilian Institute of Geography and Statistics, the elderly population grew 47.8% in the last decade in Brazil. A portion of this population has severe and/or profound hearing loss and do not benefit from conventional hearing aids. Thus, the use of cochlear implant is required.

**Aim:**

To analyze the benefits of cochlear implants in the elderly based on the comparison of primary auditory thresholds before and after the operation, discrimination of sentences in speech and in talking on the telephone.

**Methodology:**

Retrospective cohort study, analyzing medical records from patients aged over 60 years, users of cochlear implant for at least 1 year.

**Results:**

Fourteen medical records were analyzed. Mean age of patients was 63.07 years. The mean pure tone thresholds between 500Hz, 1kHz, 2kHz and 4kHz before the implantation was 113dBHL. None of the patients, before operation, could discriminate sentences in open sets and only 3 scored 17% in closed sets sentence recognition. After one year of implantation, the mean sound field thresholds reached 34dBHL, and open set sentences recognition of 93.57%, while 71% of the patients had become able to have a conversation on the telephone.

**Conclusion:**

The elderly users of cochlear implant showed important outcomes, with significant improvement in understanding in the open set and in using the telephone.

## INTRODUCTION

According to current IBGE data, the Brazilian population aged 60 years and over has increased 47.8% in the last decade; it is now 10.5% of the country's population.^1^

This increased in the elderly population brings new demands for quality of life improvements and adaptation of this group into the labor market.

Hearing loss is one of the most frequent difficulties for this age group, and probably one of the most incapacitating. Hearing loss leads to several problems such as social withdrawal, loneliness and depression, which may seriously affect the quality of life.^2^^,^^3^ Additionally, elderly persons become dependent of other for daily activities such as answering the telephone, going out, and watching television, among others.

Conventional hearing aids effectively treat different degrees of deafness,^4^ except for cases of severe and/or profound hearing loss, in which even the most powerful hearing aids may not solve the auditory needs of these patients. There is not enough cochlear reserve to attain auditory thresholds for discriminating speech sounds and to understand open sentences; thus, the option in such cases is the cochlear implant.^5^

Cochlear implants are surgically implanted devices that replace the function of injured or absent hair cells to produce an electrical stimulus for the remaining fibers of the auditory nerve.^6^

The purpose of this study was to assess the benefits of cochlear implants in the elderly, as this age group is increasing in Brazil but is not often mentioned in cochlear implant studies.^7^

## MATERIALS AND METHODS

This cross-sectional retrospective study consisted of analyzing the files of patients aged over 60 years that had used cochlear implants for at least one year. Patients aged below 60 years on the date of surgery, and patients that had used cochlear implants for less than one year were not included in this study.

Table 1 shows the demographic data and the characteristics of deafness in the sample.

**Table 1:** Demographic data of the study sample.

Subject	Age at surgery (years)	Sex	Duration of deafness (years)	Time of use of the implant (years)	Etiology	Model of the implant
1	60	M	10	7	Unknown	N22
2	73	M	2	9	Unknown	N22
3	61	F	20	9	Unknown	N22
4	63	M	7	2	Unknown	N24
5	66	M	3	4	Intoxication by bee sting	N24
6	60	M	3	8	Unknown	N22
7	65	F	4	2	Unknown	N24
8	62	F	5	8	Unknown	N22
9	66	F	5	6	Unknown	N24
10	67	F	4	4	Cranial trauma	N24
11	60	F	15	6	Unknown	N24
12	60	M	17	6	Cranial trauma	N24
13	60	F	20	6	Unknown	N24
14	60	M	18	4	OMC	N24

Patients used the Cochlear Nucleus 22K (N22) and Cochlear Nucleos 24K (N24) multichannel cochlear implant devices.

The preoperative and the 12-month cochlear implant use postoperative assessments were analyzed. The preoperative assessments were done with the patients using the hearing aids they had before surgery. The postoperative assessment was done with the speech processor of the cochlear implant starting at three months of use of this device.

A Madsen Midimate 622 audiometer connected to loudspeakers through an Orlandi acoustic amplifies was used for free field audiometry.

Speech perception tests were done according to the following protocol: Ling's sound detection and discrimination; discrimination of vowels; supra-segment pattern recognition test; four-choice test; sentence recognition test (closed); sentence recognition test (open); monosyllable recognition test, and the use of telephones.

The institutional review board approved this study (protocol no. 1039/08). No free informed consent form was applied because the subject's identity was not divulged.

## RESULTS

Fourteen files were analyzed. There were no complications of surgery in the sample. The speech processor was activated one month after surgery. All 22 intracochlear electrodes in the Nucleus devices were activated in the programming of patients.

All patients used the implant during at least 8 hours daily.

**Figure 1:**
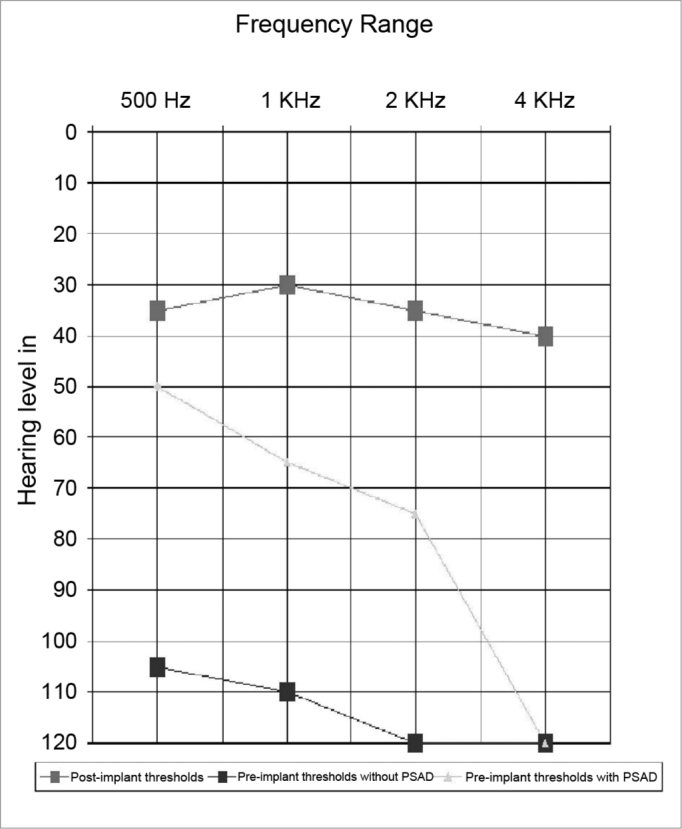
Pre- and post-implant results of free-field audiometry.

The mean pure tone threshold at 500 Hz, 1 kHz, 2 kHz and 4 kHz before implantation was 113 dBHL (minimum – 105 dB and maximum – 125 dB). The mean free field threshold with hearing aids was 78 dBHL (minimum – 45 dB and maximum – 110 dB) (Fig. 1).

The mean rates of right answers were: vowel discrimination (30%), the four-choice test (38%) and the monosyllable recognition test (4%) when using hearing aids (Table 2).

**Table 2:** Mean pre- and post-implant speech perception (in % of right answers) in the study population.

	Vowels	Four-choice	Monosyllables	Sentences closed context	Sentences open context
Pre-implant (%)	30	38	04	03	0
Post-implante (%)	99,5	97,8	61	100	93

Before the implants, no patient had been able to discriminate sentences in an open context, and only 3 were able to do it in a closed context (with a 17% rate of right answers). Similarly, none of them were able to have telephone conversations.

The mean free-field threshold one year after implant use reached 34 dBHL, as seen in Figure 1.

The mean rate of right answers in vowel discrimination reached 99.5%; it was 97.8% in the four-choice test and 61% in monosyllable recognition (Table 2).

**Figure 2:**
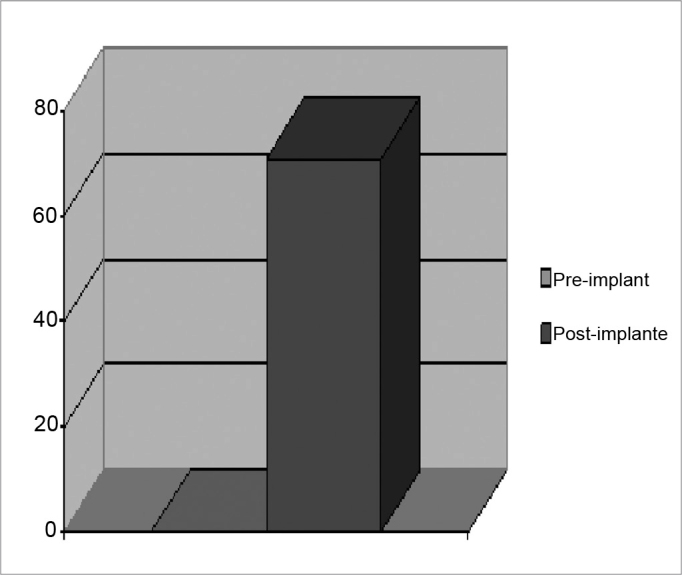
Percentage of patients using telephones before and after cochlear implant use.

Sentence discrimination in an open context reached 93.57% (Table 2). Telephone conversations became possible for 71% of patients (Fig. 2).

## DISCUSSION

Elderly patients comprise the age group with the lowest rate of procedures (3.33%) among all patients undergoing cochlear implant surgery in our group.

This is mainly due to misgivings about surgery, which is generally seen in elderly persons and their family members. Patients and healthcare professionals also lack information about cochlear implants, which also explains the paucity of this procedure.

The results of elderly patients using cochlear implants in our group showed relevant gains in hearing, significant improvement in speech recognition in open contexts, and routine telephone use.

Pasanisi et al. (2003^8^) studied 16 elderly patients that used cochlear implants and found that, 12 months after surgery, there were no significant performance differences between the study and control groups. Djalilian et al. (2002^9^) analyzed 31 elderly patients that used cochlear implants and found a major improvement in the audiometric thresholds, similar to patients under the age 60 years.

Orabi et al. (2006^10^) demonstrated that there were major benefits in speech perception tests and in the quality of life of elderly patients with implants; they based their assessment on tests and quality of life questionnaires applied after surgery (the Glasgow Benefit Inventory or GBI, and the Glasgow Health Status Inventory Questionnaire or GHSI). Vermeire et al. (2005^11^) also applied these tools in a study of 25 elderly users of cochlear implants, and found that there were significant benefits in their quality of life, similar to that in young users.

Eventual neural degeneration aspects^12^, ^13^, ^14^, ^15^ and inefficient central auditory processing in elderly subjects^16^ should be taken into account in the preoperative assessment and during adaptation of expectations before cochlear implant surgery. These factors may also affect how messages are understood after cochlear implants are placed.

Still, as shown in our results, it is clear that age should not be a relevant or excluding factor when choosing candidates for surgery.

## CONCLUSION

Elderly patients using cochlear implants showed relevant auditory gains and significant improvements in their open context understanding and telephone use.
